# Neutralization of LINGO-1 during *In Vitro* Differentiation of Neural Stem Cells Results in Proliferation of Immature Neurons

**DOI:** 10.1371/journal.pone.0029771

**Published:** 2012-01-03

**Authors:** Camilla Lööv, Maria Fernqvist, Adrian Walmsley, Niklas Marklund, Anna Erlandsson

**Affiliations:** 1 Department of Neuroscience, Uppsala University, Uppsala, Sweden; 2 Autoimmune, Transplantation & Inflammation DA, Novartis Institutes for Biomedical Research, Basel, Switzerland; Institut de la Vision, France

## Abstract

Identifying external factors that can be used to control neural stem cells division and their differentiation to neurons, astrocytes and oligodendrocytes is of high scientific and clinical interest. Here we show that the Nogo-66 receptor interacting protein LINGO-1 is a potent regulator of neural stem cell maturation to neurons. LINGO-1 is expressed by cortical neural stem cells from E14 mouse embryos and inhibition of LINGO-1 during the first days of neural stem cell differentiation results in decreased neuronal maturation. Compared to neurons in control cultures, which after 6 days of differentiation have long extending neurites, neurons in cultures treated with anti-LINGO-1 antibodies retain an immature, round phenotype with only very short processes. Furthermore, neutralization of LINGO-1 results in a threefold increase in βIII tubulin-positive cells compared to untreated control cultures. By using BrdU incorporation assays we show that the immature neurons in LINGO-1 neutralized cultures are dividing neuroblasts. In contrast to control cultures, in which no cells were double positive for βIII tubulin and BrdU, 36% of the neurons in cultures treated with anti-LINGO-1 antibodies were proliferating after three days of differentiation. TUNEL assays revealed that the amount of cells going through apoptosis during the early phase of differentiation was significantly decreased in cultures treated with anti-LINGO-1 antibodies compared to untreated control cultures. Taken together, our results demonstrate a novel role for LINGO-1 in neural stem cell differentiation to neurons and suggest a possibility to use LINGO-1 inhibitors to compensate for neuronal cell loss in the injured brain.

## Introduction

Several important breakthroughs during recent years have raised a hope that stem cell-based therapies could be used to restore function and integrity after acute brain injury and other disorders of the central nervous system. In order to develop effective and safe regenerative treatments it is however necessary to identify factors that could be used to control differentiation, proliferation and survival of neural stem and progenitor cells (NSPCs). In addition to intrinsic regulation, the presence of different extrinsic factors including soluble compounds, membrane bound molecules and extracellular matrix has been shown to influence NSPCs in various ways. For example fibroblast growth factor (FGF2) [1], [2], epidermal growth factor (EGF) [3], [4], Notch [5] and sonic hedgehog (SHH) [6] all promote proliferation and prevent differentiation of NSPCs. Ciliary neurotrophic factor (CNTF), bone morphogenic protein (BMP) and leukemia inhibitory factor (LIF) has been demonstrated to shift the differentiation of NSPCs into an astrocytic fate [2], [7] whereas addition of tri-iodothyronine (T3) or insulin-like growth factor 1 (IGF-1) increase the number of oligodendocytes in NSPC cultures [2], [8]. Neuronal-specific induction is more difficult to achieve. Activation of the Wnt pathway has been demonstrated to direct neural cortical progenitor cells to differentiate to neurons *in vitro* and to promote hippocampal neurogenesis *in vivo* but the Wnt ligands has also been shown to induce proliferation of neural stem cells [9], [10], [11], [12], [13], [14]. Platelet derived growth factor (PDGF) was earlier suggested to be involved in neuronal differentiation, but has more recently been shown to rather promote proliferation of precursor cells [15], [16], [17].

Leucine rich repeat and Ig domain containing Nogo receptor interacting protein-1 (LINGO-1) is a nervous system-specific transmembrane protein that is associated with the Nogo-66 receptor complex known to be a potent inhibitor of axonal sprouting and myelination [18], [19], [20], [21], [22]. In addition, LINGO-1 has been shown to negatively regulate the differentiation of oligodendrocyte precursor cells (OPCs) to myelinating oligodendrocytes [23]. Results from both cell culture experiments and animal studies provide evidence that blocking endogenous LINGO-1 by LINGO-1 antagonists or gene knockouts promote oligodendrocytic differentiation, axonal integrity and remyelinisation in experimental models of multiple sclerosis [23]. Furthermore, it has been suggested that LINGO-1 inhibition increase neuronal survival by activation of the PI3K/Akt pathways [24]. The role of LINGO-1 for neural stem cell regulation has however not previously been evaluated. In the present study we demonstrate a function of LINGO-1 in neuronal differentiation of NSPCs.

## Results

### LINGO-1 expression increases during neural stem cell differentiation

**Figure 1:**
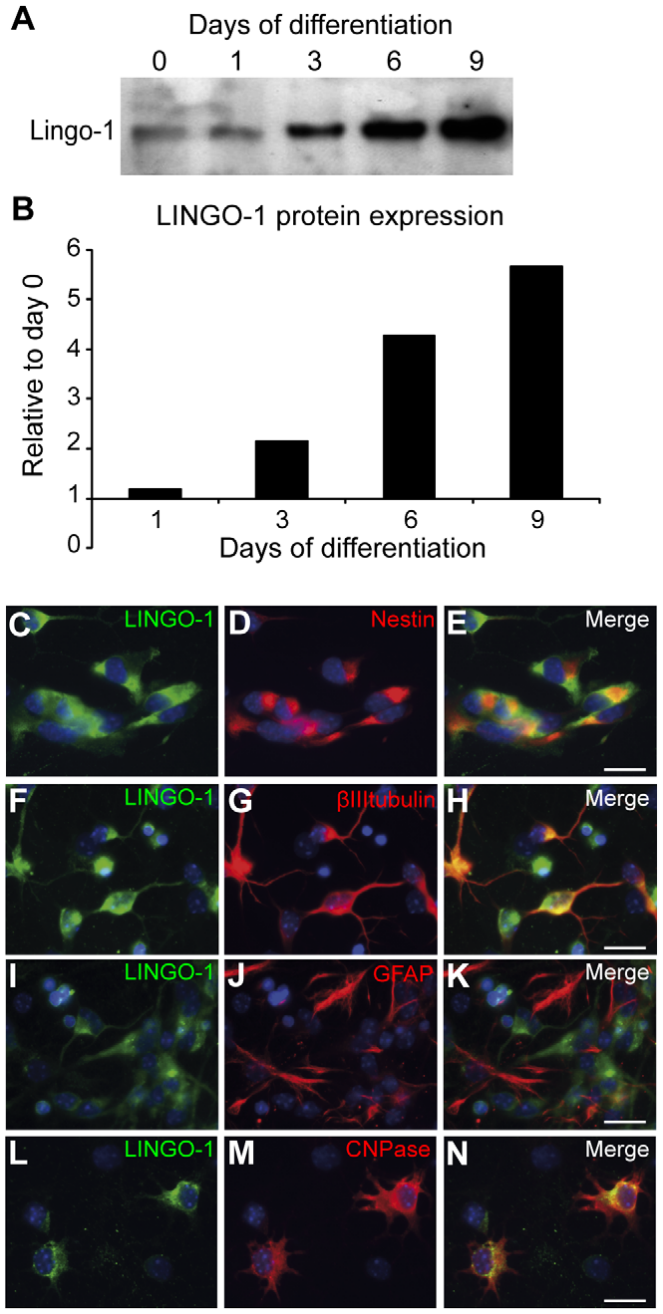
LINGO-1 expression increases during neural stem cell differentiation. A) Western blot analysis was used to study LINGO-1 expression during NSPC differentiation. Cell lysates from proliferating cells (Day 0) and NSPCs differentiating for 1–9 days was immunoprecipitated with anti-LINGO-1 antibodies (Novartis) and blotted with LINGO-1 ab (Abcam). LINGO-1 is present in both proliferating and differentiated NSPCs, but the expression increases during the differentiation. B) Protein quantification show a 9-fold increase in LINGO-1 expression in cultures differentiated for 9 days compared to proliferating NSPCs (Day 0). Double immunostainings with specific antibodies against LINGO-1 (C–N) and nestin (C–E), βIIItubulin (F–H), GFAP (I–K) or CNPase (L–N) show that NSPCs, neurons and oligodendrocytes express LINGO-1, but not astrocytes. The NSPC cultures were fixed at day 0 (C–E) and the differentiated cultures were fixed at day 6 after mitogen withdrawal (F–N). Scale bars = 20 µm.

Western blot analysis was used to investigate the expression of LINGO-1 during NSPC differentiation. Cell lysates were prepared from NSPCs proliferating in the presence of the mitogens EGF and FGF2 and from NSPCs that have differentiated in the absence of the mitogens for 1, 3, 6 and 9 days. The lysates were immunoprecipitated with a LINGO-1 specific antibody (LINGO-1 ab) and following transfer, the membrane was hybridized with another LINGO-1 specific antibody. Figure 1A show that LINGO-1 is present in proliferating, undifferentiated NSPCs (Day 0) although the protein level is low. The expression of LINGO-1 increases as the cells differentiate and the maximum expression of LINGO-1 was detected in lysates from cells that have differentiated for the longest time (Day 9). Quantification of the LINGO-1 expression show a nine-fold increase in the expression at 9 days of differentiation compared to Day 0 (Figure 1B).

In order to investigate the expression of LINGO-1 in specific cell types during NSPC differentiation we performed double immunostainings using antibodies against LINGO-1 and specific markers for NSPCs, neurons, oligodendrocytes and astrocytes. Proliferating NSPCs were fixed at day 0 and stained with antibodies against nestin and LINGO-1. We found that 91±1% (mean±sem) of the cells at day 0 were nestin positive and that 100±0% (mean±sem) of these nestin-positive NSPCs expresses LINGO-1 (Figure 1C–E). Differentiated cultures were fixed 6 days after growth factor withdrawal and stained with antibodies against LINGO-1 and ßIII tubulin (neurons), CNPase (oligodendrocytes) or GFAP (astrocytes). In line with previous studies [18], [20], [23], our immunostainings demonstrate that 100±0% (mean±sem) of both the neurons and oligodendrocytes, but 0±0% (mean±sem) of the astrocytes, express LINGO-1 (Figure 1F–N). In order to test the specificity of the LINGO-1 antibody we performed performed double stainings with the Novartis antibody and a LINGO-1 antibody purchased from Abcam. The staining demonstrates that the two antibodies identify the same LINGO-1 expressing cells in the culture (Figure S1).

### Neurons in LINGO-1 neutralized cultures retain an immature phenotype

**Figure 2:**
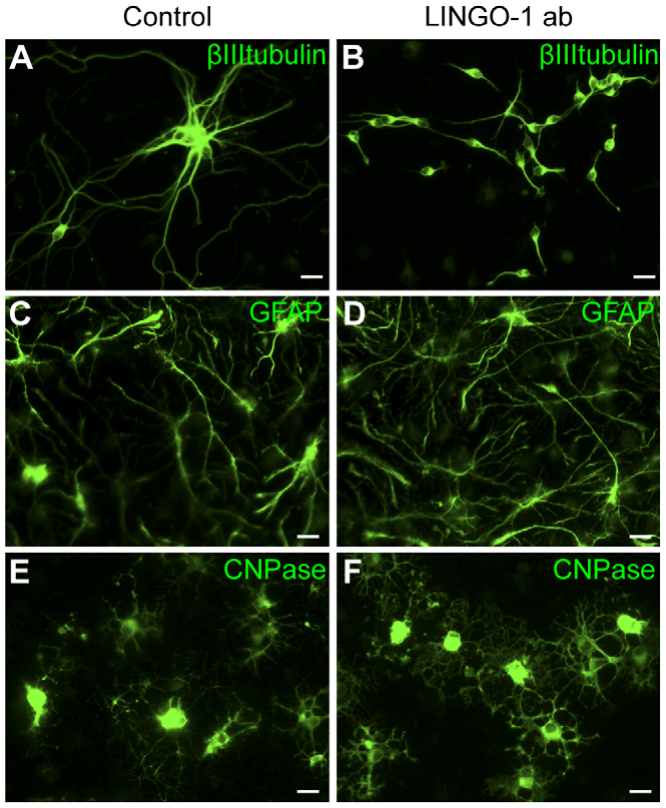
LINGO-1 neutralization during NSCP differentiation results in immature neurons. To investigate the effect of LINGO-1 neutralization on NSPC differentiation, NSPCs were cultured for 6 days in the absence (A, C and E) or presence (B, D and F) of LINGO-1 ab (Novartis) following mitogen withdrawal. The cells were fixed and stained with specific antibodies against βIIItubulin (A–B), GFAP (C–D) or CNPase (E–F). In control cultures (A) neurons were rather mature with multiple, long extending processes, but in cultures treated with LINGO-1 antibodies (B) the neurons had a more immature phenotype with only one or two short processes. There was no distinct difference in astrocyte staining (C–D) or oligodrocyte staining (E–F) between control cultures and LINGO-1 inhibited cultures. Scale bars = 20 µm.

Our western blot data show that LINGO-1 is expressed in NSPCs, but that the expression increases during the differentiation. We next sought to investigate the effect of LINGO-1 neutralization on NSPC differentiation. Differentiation of NSPC cultures was initiated by mitogen removal and cells were cultured in medium only (controls) or medium containing 100 µg/ml anti-LINGO-1 antibodies (LINGO-1ab) for 1, 3 or 6 days prior to fixation. Parallel cell cultures were immunostained against markers specific for neurons (βIII tubulin), astrocytes (GFAP) and oligodendrocytes (CNPase) (Figure 2). Our results show that neutralization of LINGO-1 has a dramatic effect on neuronal differentiation (Figure 2A, B). Compared to βIII tubulin-positive cells in untreated control cultures, which after 6 days of differentiation have a rather mature neuronal phenotype with long extending neurites (Figure 2A), βIII tubulin-positive cells in cultures treated with LINGO-1 ab retain an immature, round phenotype with only very short processes (Figure 2B). In contrast, astrocyte differentiation was not noticeably influenced by the neutralization of LINGO-1 as GFAP-positive cells in control cultures (Figure 2C) and cultures treated with LINGO-1 ab had identical phenotypes. Furthermore, we found that CNPase-positive oligodendrocytes appeared only slightly more differentiated after 6 days when cultured in the presence of LINGO-1 ab compared to untreated controls (Figure 2E, F). Our results show that LINGO-1 is especially important for early neuronal differentiation and that neutralization of LINGO-1 result in decreased neuronal maturation. To verify that the effect of the LINGO-1 neutralization was specific, a control antibody (100 µg/ml anti-lysozyme) was included as a control (Figure S2). Since the effect of the control antibody was indistinguishable from plain medium, untreated cultures was used as controls in all additional experiments. Furthermore, we performed experiments with different concentrations of the LINGO-1 antibody. We found that already at the lower concentrations, 1 µg/ml and 10 µg/ml, we had a clear affect of the LINGO-1 antibody on neuronal maturation (the neurons had shorter processes). The effect was however more pronounced in cell cultures treated with 100 µg/ml LINGO-1 ab. The effect on neuronal differentiation in cultures treated with 1000 µg/ml LINGO-1 antibody was similar to 100 µg/ml, but the cells were more often found in clusters (Figure S2).

### Neutralization of LINGO-1 results in an increased number of neurons

**Figure 3:**
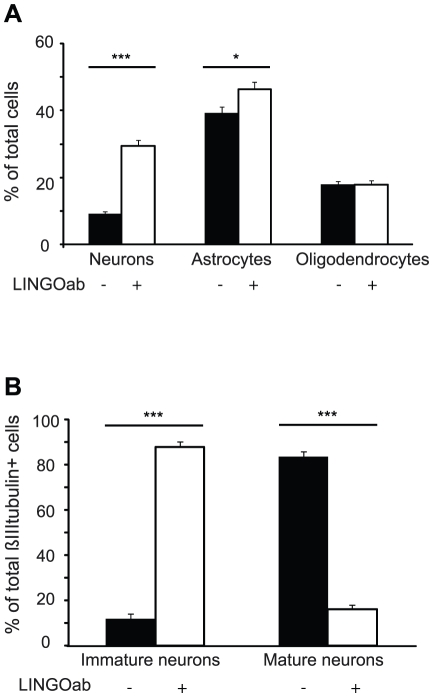
Neutralization of LINGO-1 leads to an increased percentage of neurons. A) The number of neurons, astrocytes and oligodendrocytes was counted and plotted as the ratio of specific marker-positive cells to the total cell number (DAPI). After 6 days of differentiation there was a 3-fold increase in the percentage of neurons in LINGO-1 ab treated cultures compared to untreated controls. There was only a modest, but significant, increase in the percentage of GFAP positive cells and no difference was found in the percentage of CNPase positive cells. B) Comparison of the percentage of mature and immature neurons of the total number of βIIItubulin positive cells show a 7-fold increase of immature cells in LINGO-1 ab treated cultures compared to untreated control cultures after 6 days of differentiation. *** denotes p<0,001, ** denotes p<0,01 and * denotes p<0,05.

To elucidate if the percentage of neurons, astrocytes and oligodendrocytes in the differentiated NSPC cultures were influenced by LINGO-1 neutralization, we counted the βIII tubulin, GFAP and CNPase-positive cells in control cultures after 6 days of differentiation in the absence or presence of LINGO-1 ab. The percentage of positive cells to the total cell number is presented in Figure 3A. We noted a 3-fold increase of βIII tubulin positive cells in LINGO-1 neutralized cultures (29.5±0.7% mean±sem) compared to control cultures (9.1±1.6% mean±sem) (Figure 3A). There was a modest, but significant, increase in the percentage of GFAP-positive cells in LINGO-1 neutralized cultures compared to untreated control cultures, but no difference in the percentage of CNPase-positive cells (Figure 3A). We have shown that astrocytes do not express LINGO-1. It is however probable that early astrocytic progenitor cells express the LINGO-1 which could explain the increase of GFAP-positive cells in LINGO-1 neutralized cultures. Taken together, the morphology of the different cell types shown in Figure 2 and the cell counting experiments shown in Figure 3 demonstrate that the neutralization of LINGO-1 during early NSPC differentiation has a clear effect on neuronal maturation but only a mild effect on glial maturation. We therefore decided to focus on neuronal maturation in this study.

Next, we compared the percentage of mature and immature neurons in control cultures and cultures treated with LINGO-1 ab after 6 days of differentiation (Figure 3B). Neurons with multiple, long extending processes were considered to be mature while neurons with only one or two short processes and a round cell body was considered to be immature. We found a striking difference between the cultures. The percentage of immature neurons in untreated control cultures was 12.0±2.2% (mean±sem) compared to 83.9±1.8% (mean±sem) in cultures that received LINGO-1 ab during the differentiation period. The percentage of mature neurons showed the opposite pattern with 88.0±2.2% (mean±sem) in the control cultures compared to 16.1±1.8% (mean±sem) in cultures treated with the antibody. The 7-fold increase of immature cells in LINGO-1 neutralized cultures further demonstrates the important role of LINGO-1 in the differentiation of NSPCs into neurons.

### Cell proliferation is increased in LINGO-1 neutralized stem cell cultures

We next examined if neutralization of LINGO-1 influences the proliferation of NSPCs. We first investigated the effect of LINGO-1-blocking on the ability of the NSPCs to form neurospheres in the presence or absence of the mitogens FGF2 and EGF. NSPC neurosphere cultures were dissociated to single cell suspension and 10 NSPCs/µl was cultured for 8 days in medium containing LINGO-1 ab, LINGO-1 ab+FGF2+EGF and control cultures with FGF2+EGF or medium only. Neurospheres were only found in cultures with FGF2 and EGF and there was no significant difference in neurosphere number between the cultures with both mitogens and LINGO-1 (203±3 spheres/ml mean±sem) and mitogens only (191±13 spheres/ml mean±sem) indicating that LINGO-1 neutralization does not affect proliferation of immature, sphere forming neural stem cells.

**Figure 4:**
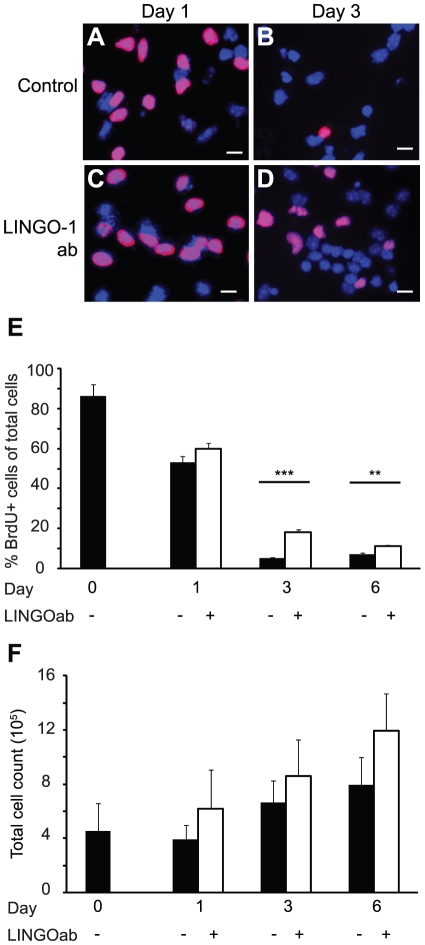
LINGO-1 neutralization promotes cell proliferation. BrdU labeling was used to investigate the effect of LINGO-1 neutralization on cell proliferation (A–D). Cells were exposed to BrdU for 16 hours prior to fixation and stained with specific antibodies against BrdU (red) and DAPI (blue). E) The number of cells that had incorporated BrdU was counted and plotted as the ratio of BrdU-positive cells to the total cell count. F) The total cell number in anti-LINGO-1 antibody treated and untreated control cultures were measured at day 0, 1, 3 and 6 days of differentiation. The cells in each dish were harvested using a cell scraper and counted using a NucleoCounter™. The mean values of the total cell count/dish were plotted. *** denotes p<0,001, ** denotes p<0,01 and * denotes p<0,05 and scale bars = 20 µm.

To investigate whether LINGO-1 inhibition increase DNA synthesis of progenitor cells during the first days of differentiation, cell cultures were pulse-labeled with bromodeoxyuridine (BrdU) for 16 hours and stained with an anti-BrdU antibody (Figure 4A–E). The BrdU incorporation in NSPC cultures grown in the presence of FGF and EGF was 87.5±5.6% (mean±sem) (Day 0, Figure 4A). In control cultures, differentiating in the absence of growth factors, the percentage of cells that had incorporated BrdU decreased markedly and after three days of differentiation only 5.0±0.5% (mean±sem) were BrdU positive (Figure 4A–C). After one day of differentiation, cultures treated with LINGO-1 ab had no significant increase in BrdU incorporation compared to untreated control cultures, but after 3 days of differentiation the increase in BrdU incorporation was 3-fold (18.0±1.6% mean±sem, Figure 4A, D–E). At day 6, the proliferation had declined in the LINGO-1 neutralized cultures, but there was still a significantly higher BrdU incorporation compared to the untreated control cultures (11.0±0.8 and 7.0±0.8% mean±sem respectively, Figure 4A).

To further clarify the mitogenic effect of LINGO-1 neutralization during the first days of NSPC differentiation, we investigated if treatment with LINGO-1 ab resulted in an increase in the total cell number. For this purpose all the cells in the dish of LINGO-1 neutralized cultures and control cultures were collected by using a cell scraper. The total number of cells was counted by using a nuclear cell counter after 1, 3 and 6 days of differentiation (Figure 4F). We found that neutralization of LINGO-1 did not significantly increase the total cell number (Figure 4F). This could be explained by the fact that only a low percentage of progenitor cells are proliferating in the differentiating cell cultures, making the result of a 3-fold increase in BrdU positive cells difficult to detect in the total cell number. Taken together our results demonstrate that blocking LINGO-1 has no direct effect on neural stem cell expansion, but probably increase proliferation of one or several types of neural progenitor cells.

### LINGO-1 neutralization specifically increase proliferation of neuroblasts

**Figure 5:**
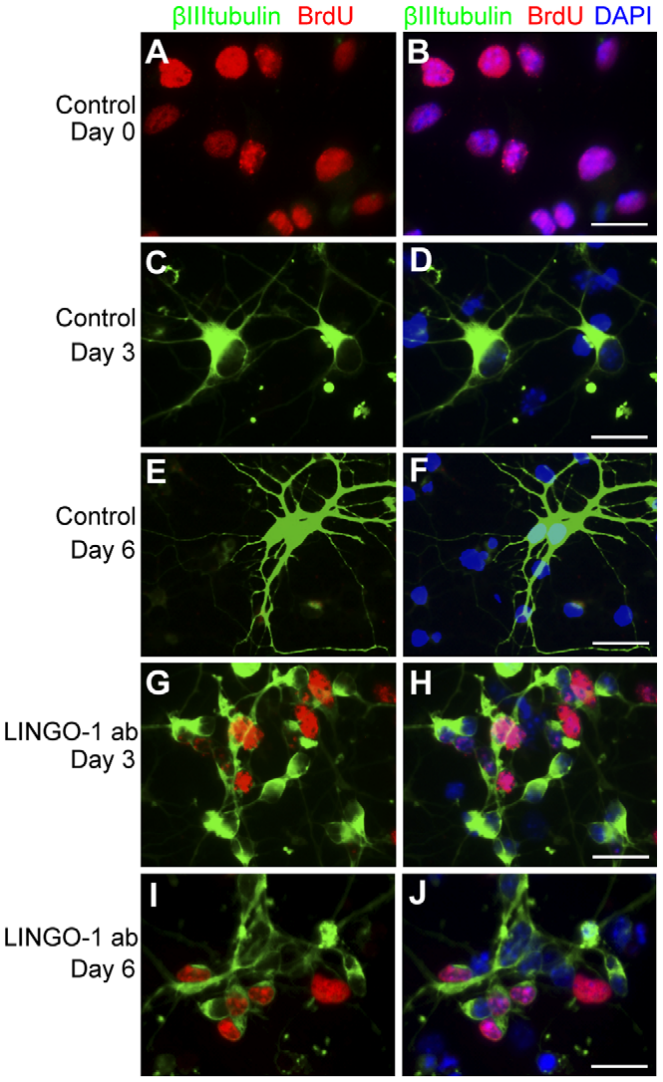
Neutralization of LINGO-1 leads to an increased percentage of proliferating neuroblasts. To investigate if the immature neurons in LINGO-1 neutralized cultures proliferate, we double-labeled the cells with specific antibodies against BrdU (red), βIIItubulin (green) and DAPI (blue). NSPCs were fixed at day 0 (A–B) or differentiated in only medium for 3 days (C–D) or for 6 days (E–F) medium containing anti-LINGO-1 antibodies (G–J). BrdU was added to the cultures 16 hours prior to fixation. Scale bars = 20 µm.

Our immunostainings show that neuroblasts do not differentiate into mature neurons during 6 days of differentiation in the presence of LINGO-1 ab. To investigate if LINGO-1 neutralization has a specific effect on the proliferation of immature neurons, NSPCs were differentiated in the absence or presence of LINGO-1 ab, pulse-labeled with BrdU for 16 hours and stained with antibodies against BrdU and βIII tubulin after fixation (Figure 5A–J and Table 1). As we previously showed in Figure 4E, a very high proportion of the NSPCs were BrdU-positive at the beginning of the experiment, but these cells did not express neuronal markers (Figure 5A–B and Table 1). Already after 3 days of differentiation, the cells in the control cultures that expressed the neuronal marker had a rather mature phenotype. We did not find any cells that were double positive for βIII tubulin and BrdU, demonstrating that the cells that had differentiated to neurons ceased to divide (Figure 5C–D and Table 1). At day 6 after growth factor withdrawal, the neurons were more mature, with several long, extending processes, but also here devoid of BrdU incorporation (Figure 5E–F and Table 1). In cultures treated with LINGO-1 ab the results were different. After 3 days of differentiation 35.5±4.1% (mean±sem) of the cells expressing the neuronal marker βIII tubulin were also positive for BrdU, showing that LINGO-1 neutralization has a prominent effect on neuroblast proliferation (Figure 5G–H and Table 1). After 6 days of differentiation the percentage of proliferating immature neurons had declined, but still 13.4±3.3% (mean±sem) of the neurons had incorporated BrdU (Figure 5I–J and Table 1).

**Table 1:** LINGO-1 neutralization increases the percentage of proliferating immature neurons.

	Day 0	Day 3	Day 6
**Control** a	0%±0%	0%±0%	0%±0%
**LINGO-1 inhibition** a	N/A	35.5%±4.1%***	13.4%±3.3%***

a The number of BrdU-βIIItubulin double positive cells and total number of βIIItubulin positive cells were counted and the proportion of dividing neurons calculated. The results are expressed as means ± standard error of the mean (sem). *** = p<0.001.

### LINGO-1 neutralization increase cell survival of differentiating neural stem cells

**Figure 6:**
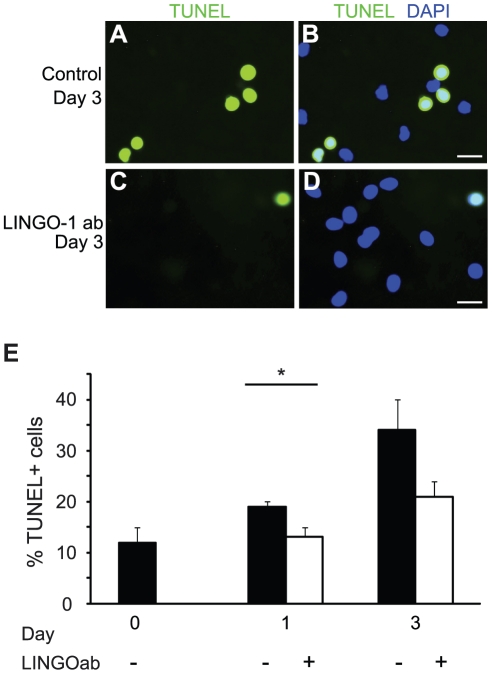
Decreased number of TUNEL positive cells in cultures treated with anti-LINGO-1 antibodies. TUNEL assay was performed on NSPC and parallel cultures of cells differentiated for 1 and 3 days in the absence or presence of LINGO-1 ab (A–D). Representative photos of control cultures (A–B) and LINGO-1 neutralized cultures (C–D) at day 3 of differentiation, TUNEL (green) and DAPI (blue). E) Cells going through apoptosis were counted and plotted as the ratio of the total number of cells. *** denotes p<0,001, ** denotes p<0,01 and * denotes p<0,05 and scale bars = 20 µm.

**Figure 7:**
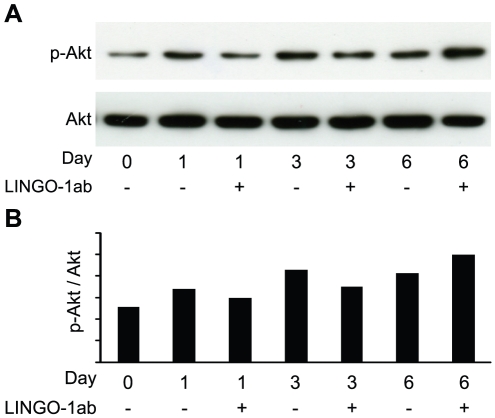
LINGO-1 neutralization has no effect on PKB/c-Akt phosphorylation. A) Western blot analysis was used to study PKB/c-Akt phosphorylation in differentiating NSPCs in the absence or presence of LINGO-1 ab. Total cell lysates were used for immunoblotting with anti-PKB/c-Akt antibody (Akt) and anti-phosphorylated PKB/c-Akt antibody (p-Akt). B) The ratio of phosphorylated Akt was measured and plotted.

We next investigated if inhibition of LINGO-1, in addition to the effect on proliferation, also has an effect on cell survival during the first days of NSCP differentiation. Staining for dead cells using TUNEL labeling, revealed a significant decrease in cell death in LINGO-1 ab-treated cultures compared to control cultures already after 1 day of differentiation. After 3 days of differentiation there was an almost 2-fold decrease in TUNEL positive cells in the LINGO-1 neutralized cultures compared to control cultures (Figure 6A–E). In addition to the TUNEL assay, we studied the phosphorylation of PKB/c-Akt as a measurement of increased cell survival since LINGO-1 neutralization previously has been indicated to result in a sustained Akt-phosphorylation in retinal ganglion cells [24]. We measured phosphorylated and total PKB/c-Akt in protein lysates from parallel cell cultures differentiating in the absence or presence of LINGO-1 ab for 1, 3 and 6 days by Western blot. The highest level of phosphorylated PKB/c-Akt was found in cultures differentiated for six days in the presence of LINGO-1 ab. We could however not detect any clear differences in PKB/c-Akt phosphorylation between LINGO-1 ab-treated cultures and control cultures at the different time points (Figure 7A–B).

## Discussion

Here we report a novel function for LINGO-1 in neural stem cell differentiation, regulating the maturation of progenitor cells differentiating along the neuronal lineage. Neutralization of LINGO-1 during the first days of neural stem cell differentiation results in a prominent decrease in neuronal maturation. Compared to neurons in control cultures, which after 6 days of differentiation have long extending neurites, neurons in cultures treated with LINGO-1 ab retain an immature, round phenotype with only very short processes. It has previously been reported that LINGO-1 is a negative regulator of differentiation of OPCs to mature, myelinating oligodendrocytes. Treatment of cultured OPCs with LINGO-1 siRNA, dominant negative LINGO-1 or LINGO-Fc, resulted in increased morphological differentiation of the oligodendrocytes characterized by the abundance of terminal membrane sheets [23]. In another study nerve growth factor (NGF) was shown to regulate the expression of axonal LINGO-1 and thereby the inhibition of oligodendrocyte differentiation and myelinisation [20]. In contrast to these findings, that demonstrate an important role for LINGO-1 during late oligodendrocyte maturation, we only detected minor effects of LINGO-1 neutralization on early astrocyte and oligodendrocyte maturation. Taken together, our results show that LINGO-1 is central for the regulation of early neuronal maturation, but might be less important for the glial cell fate during the first days of NSPC differentiation. Importantly, it has been demonstrated in a previous study that Myt1l, an early marker for postmitotic neurons and a neurogenic transcription factor known to enhance the maturation rate of neurons, interacts with LINGO-1 [25].

LINGO-1 expression has been detected in the developing mouse brain from day E16, and the level of LINGO-1 protein was shown to increase from late embryogenesis to peak at P5 in the cerebellum and at P21 in the anterior brain [25]. The components of the Nogo receptor complex; LINGO-1, NgR1, p75^NTR^ and TROY has been shown to coexist in the mouse brain at later postnatal stages. However, the authors show that LINGO-1 is expressed earlier during the development in the absence of NgR1, indicating that LINGO-1 therefore may participate in other activities in developing neurons separate from oligodendrocyte maturation or axon extension [25]. More recently, Mathis et al. demonstrated that migrating neural progenitor cells cultured from the E15.5 mouse brain express both the Nogo receptor, LINGO-1, TROY and p75^NTR^ [26]. In the adult brain LINGO-1 protein levels are highest in hippocampus, neocortex and striatum, while lower levels of LINGO-1 protein are found in cerebellum, pons, olfactory bulb and spinal cord [25]. It has been suggested in several reports that LINGO-1 mRNA is expressed in neurons and oligodendrocytes, but not in astrocytes [18], [20], [23]. In a detailed analysis of LINGO-1 expression in the brain, Llorens et al. found LINGO-1 protein expression in a subset of neurons, but not in myelinating, mature oligodendrocytes [25]. Moreover, Satoh et al. reported that LINGO-1 is expressed in reactive astrocytes and microglia in human brain tissue from multiple sclerosis (MS) patients [27]. Our data demonstrate that LINGO-1 is expressed by cortical neural stem cells from E14 mouse embryos, and that the LINGO-1 protein expression increases as the stem cell cultures differentiate. In NSPC cultures that have differentiated for 6 days in the absence of EGF and FGF-2, LINGO-1 is selectively expressed by neurons and oligodendrocytes and not by astrocytes. Notably, at this time point the oligodendrocytes and neurons are not fully mature.

In this investigation we neutralized LINGO-1 using an LINGO-1 ab at a concentration of 100 µg/ml based on previous studies and our initial results that this concentration effectively neutralizes LINGO-1 without adverse effects [20], [28], [29]. To exclude any non-specific effects of the LINGO-1 ab, we included a control (anti-lysozyme) antibody of the same concentration in our first sets of experiments. Since no effect of the control antibody was detected on neuronal differentiation, we used plain medium as a control in all following experiments.

Although, the influence of exogenous factors on differentiation of NSPC has been addressed in several studies, the regulation of the neuronal lineage is still unclear. In this study we demonstrate that neutralization of LINGO-1 during the first days of NSPC differentiation result in a 3-fold increase of βIII tubulin-positive cells compared to untreated control cultures. In contrast, there was only a modest increase in the percentage of GFAP positive cells in LINGO-1 neutralized cultures compared to untreated control cultures, and no difference was found in the percentage of CNPase positive cells. By using the neurosphere assay we demonstrate that LINGO-1 neutralization had no detectable effect on the ability of neural stem cells to proliferate and form neurospheres. These results further verify that LINGO-1 is primarily involved in the regulation of neuronal differentiation.

Our BrdU incorporation analyzes show that the immature neurons that are found in LINGO-1 neutralized cultures are dividing neuroblasts. In control cultures there were no cells that were double positive for βIII tubulin and BrdU after three or six days of differentiation, demonstrating that stem cells that have started to differentiate to neurons did no longer divide. In cultures treated with LINGO-1 ab the results were completely different. After three days of differentiation, 36% of the cells that expressed the neuronal marker were proliferating. After six days of differentiation the percentage of proliferating immature neurons had declined, but still 13% of the neurons incorporated BrdU.

The myelin-associated inhibitors Nogo-A, myelin-associated glycoprotein (MAG) and oligodendrocyte myelin glycoprotein (OMgp) share two common receptors; the Nogo-66 receptor (together with its co-receptors p75^NTR^, TROY and LINGO-1) and the paired immunoglobulin-like receptor B (PirB). It is well-known that these proteins impair neuronal regeneration by limiting axonal sprouting and have received much attention as promising treatment targets for e.g. traumatic brain injury, spinal cord injury and stroke [19], [21], [28], [30], [31], [32], [33]. LINGO-1 upregulation has been demonstrated in oligodendrocyte progenitor cells in MS lesions, in dopaminergic neurons in patients with Parkinson's disease and in rat spinal cord after injury [27], [28], [32], [34]. It has been shown that mice deficient in LINGO-1 or treated with LINGO-1 neutralizing antibodies exhibit increased remyelinisation in experimental autoimmune encephalomyelitis (EAE), a model of immune-mediated demyelinisation [21]. In another study, an LINGO-1 antagonist was demonstrated to promote CNS remyelinisation by directly stimulating OPC differentiation in nonimmune, toxin-induced models of demyelination in rats [29]. Moreover, it has been shown that neutralization of LINGO-1 has a positive effect of cell survival in animal models of various CNS disorders including spinal cord injury, MS and Parkinson's disease and LINGO-1 inhibitors are currently evaluated as a treatment option for MS [19], [34], [35]. The possible effect of LINGO-1 neutralization on cell survival in differentiating NSPC cultures was investigated by TUNEL assay and Western blot analysis of phosphorylated PKB/Akt. Our TUNEL stainings revealed that the amount of cells going through apoptosis during the early phase of differentiation (1 and 3 days following growth factor withdrawal) was significantly decreased in cultures treated with LINGO-1 ab. However, there was no increase in phosphorylated PKB/Akt in LINGO-1 neutralized cultures, indicating that other cell death pathways, possibly associated with p75^NTR^ or RhoA, might be involved [34], [36].

Recent studies demonstrate that both Nogo and NgR1 are present on neural stem cells in culture [37], [38], [39]. The exact role for myelin-associated inhibitors and their receptors during neural stem cell differentiation is however still unclear. It has been reported that Nogo-66 (the axon growth inhibiting domain of Nogo-A) and MAG could promote astroglial differentiation of NSPCs [38], [40]. In another study, two other regions of Nogo-A was shown to inhibit neuronal differentiation and promote glial cell formation [41]. Furthermore, the myelin-associated inhibitor OMgp has been shown to be expressed by cultured NSPCs and over expression of OMgp in NSPC cultures had a negative effect cell proliferation [42].

Our present investigation demonstrates a striking effect of LINGO-1 neutralization on NSPCs differentiation, resulting in an expansion of the pool of immature neurons. The long-term effect of LINGO-1 neutralization, however, remains to be elucidated. It is possible that the proliferating, immature neurons eventually differentiate into mature neurons (although slower than neurons in the control cultures). Our results highlight the possibility to use LINGO-1 inhibitors in combination with stem cell transplantations or in order to promote endogenous neurogenesis following CNS insults or neurodegenerative disorders. For clinical reasons it is of course necessary to investigate if the neuroblasts that are proliferating in the presence of LINGO-1 ab give rise to functional neurons *in vivo*. Several investigations demonstrate an involvement of LINGO-1 in neurodegenerative processes including Alzheimer's disease [43], Parkinson's disease [28] and multiple sclerosis [21]. In order to develop therapeutic strategies based on LINGO-1 neutralization it is necessary to understand the basic biology of LINGO-1 and its effect on the different CNS cell types.

## Materials and Methods

### Anti-LINGO-1 antibody

To neutralize LINGO-1 during NSPC differentiation we used a human anti-LINGO-1 monoclonal antibody provided by Novartis Inc. Basel, Switzerland [44]. Based on previous cell culture studies using anti-LINGO-1 ab we tested four different concentrations; 1, 10, 100 and 1000 µg/ml [20], [28], [29]. The effect of the different concentrations was similar, but the most pronounced effect on neuronal differentiation was found using the higher concentrations, 100 µg/ml and 1000 µg/ml. In culture treated with 1000 µg/ml the cells were more often found in clusters (Figure S1). We chose to use 100 µg/ml throughout the study in order to maximize the LINGO-1 inhibition (since no side effects were observed at this concentration). Control (anti-chick lysozyme, 100 µg/ml) antibodies were included in the initial experiments. Since the control antibody had no effect compared to untreated controls, plain medium was used as a control in the rest of the study.

### Cell culture

C57/BL6 mice were housed at 24°C in 12 h light/dark cycles with access to food and water *ad libitum* in line with the Swedish animal welfare legislation. The study was specifically approved by Uppsala Animal Ethics Committee, Uppsala, Sweden (Permit number: C 234/8) before the study was started. Dissected cortices from E14 mice were dissociated in GIBCO Hank's Balanced Salt Solution (1×) supplemented with 8 mM HEPES buffer solution and 50 units of penicillin and 50 µg of streptomycin per ml (Invitrogen), hereafter only referred to as HBSS. The cell suspension was centrifuged and resolved in medium (GIBCO Dulbecco's Modified Eagle Medium (D-MEM/F12) with GlutaMAX (×1) supplemented with 50 units/ml^−1^ penicillin and 50 µg/ml^−1^ streptomycin, 8 mM HEPES buffer, 1×B-27 serum free supplement) fortified with 10 ng ml^−1^ Fibroblast Growth Factor 2 (FGF2) (Invitrogen) and 20 ng ml^−1^ natural mouse Epidermal Growth Factor (EGF) (Becton Dickinson). The cells were grown non-adherently into neurospheres and passaged every 3^rd^ to 5^th^ day by dissociation in HBSS and resuspension in new medium supplemented with mitogens at concentrations previously described. Prior to each experiment, cells were dissociated in HBSS and plated as a monolayer on coverslips coated with poly-L-ornithine (Sigma-Aldrich Inc.) and laminin (Invitrogen). The first day cells were maintained in EGF and FGF2 supplemented medium (to recover from the passage) and thereafter replaced with mitogen-free medium to initiate cell differentiation. 100 µg/ml of LINGO-1 ab was added to the cell culture media and control cultures received culture media only. Every third day half of the media was changed to fresh media with or without LINGO-1 ab. NSPCs from passage 1–3 were used for the study.

### Immunocytochemistry

Primary antibodies used in the study included: Tubulin beta III isoform (βIII tubulin, 1∶200, CHEMICON International) produced in mouse, rabbit antibody to Glial Fibrillary Acidic Protein (GFAP, 1∶400, DakoCytomation), 2′,3′-cyclic nucleotide 3′-phosphodiesterase (CNPase, 1∶500, Sigma) produced in mouse and human LINGO-1 (1∶100, Novartis, 1∶200 AbCam). Secondary antibodies (IgG) used were: AlexaFluor 488-conjugated antibody to rabbit, mouse or human (1∶200, Molecular Probes) and Cy3-conjugated mouse or rabbit antibody (Sigma-Aldrich). Cell culture coverslips were fixed in 4% paraformaldehyde (PFA) and permeabilized and blocked for 30 min in 0.1% Triton X-100 (vol/vol, Sigma) and 5% natural goat serum (NGS) (vol/vol) in PBS. For LINGO-1 stainings Triton-X-100 was excluded. Incubation of primary antibodies was performed in either RT for 1–4 h or overnight in 4°C. Coverslips were washed three times in PBS before incubation with secondary antibody for 1 h in 37°C. The samples were washed three times before mounted with Vectashield Hard Set mounting medium, with or without 4′,6-diamidino-2-phenylinodole (DAPI, Vector).

### Proliferation assay

Cells were seeded on precoated culture dishes as described above. After withdrawal of FGF2 and EGF, 100 µg/ml of LINGO-1 ab was added to the cell culture media and control cultures received culture media only. For studying cell proliferation, cells were pulsed with Bromodeoxyuridine (BrdU) (1∶1000, Amersham Cell Proliferation Kit, GE Healthcare) 16 hours before fixation at day 0, 1, 3 and 6 days after withdrawal of FGF2 and EGF. Cells were fixed in ice-cold acid ethanol and nuclease/anti-5-bromo-2′deoxyuridine (1∶100; Amersham Cell Profileration Kit) were added to coverslips and incubated for 1 h at room temperature. Coverslips were washed three times in PBS and incubated with secondary antibody (1∶200; anti-mouse Alexa Flour, Invitrogen) for 1 h. Coverslips were washed three times in PBS and mounted on glass slides with Vechtashield containing DAPI (Vector).

### Terminal Deoxynucleotidyl transferase dUTP nick end labeling assay

Cells were seeded on precoated culture dishes as described above. After withdrawal of FGF2 and EGF, 100 µg/ml of anti-LINGO-1 antibody was added to the cell culture media and control cultures received culture media only. Cells were fixed in 4% PFA at day 0, 1, 3 and 6 after withdrawal of FGF2 and EGF, and TUNEL labeled as instructed by the manufacturer (Roche Applied Science). Coverslips were mounted on glass slides with Vechtashield containing DAPI (Vector).

### Fluorescence microscopy

The glass slides were analyzed using a fluorescence microscope (AxioVision, Zeiss) with the magnitude of 20×. Eight different representative fields per glass slide were photographed and the labeled cells were counted. Different immunostainings, proliferation assay and TUNEL assay were performed in triplicates at minimum three times.

### Total cell counting

Total cell counting was made using a NucleoCounter™ (Chemometec A/S, Denmark). Cells were cultured in the presence or absence of LINGO-1 ab on precoated 35 mm cell culture plates. At day 0, 1, 3 and 6 after withdrawal of FGF2 and EGF cells were harvested from the dishes by scraping and dissociated in PBS (1∶3, Invitrogen), lysis buffer (1∶3; Chemometec) and stabilizing buffer (1∶3; Chemometec). The suspensions were vortexed and directly introduced into the NucleoCassettes™ (Chemometec) and run in the NucleoCounter™. The experiment was performed in triplicate and repeated three times.

### Sphere formation assay

To form a cell suspension with single cells, spheres were centrifuged for 3 min at 900 rpm and the pellet was dissolved in 10 ml preheated HBSS and incubated rotating at 37°C for 15 min. The spheres were centrifuged for 3 min at 900 rpm and dissociated in cell culture media. Single cells, 10 cells/µl, were cultured in media containing FGF2 and EGF and LINGO-1 ab. LINGO-1 ab were also added to the wells without FGF2 and EGF. Wells without LINGO-1 ab and with FGF2 and EGF respective without FGF2 and EGF were used as controls. The cells were incubated at 37°C for 8 days where after the number of spheres in each well was counted. The experiment was performed in triplicates.

### Western blot analysis: Immunoprecipitation

At day 0, 1, 3 and 6 after withdrawal of FGF2 and EGF the media was removed and protein lysates preparation was made. Lysis buffer (20 mM Tris pH 7.5, 0.5% Triton-X-100, 0.5% Deoxycholic acid, 150 mM NaCl, 10 mM EDTA, 30 mM NaPyroP) supplemented with 15% protease inhibitor cocktail (Roche) and sodium orthovanadate (Na_3_VO_4_) (0.1 M, Sigma) was added to the Petri dishes and the cells were removed by scraping. The cell lysates were kept on ice for 30 min followed by centrifugation at 12000× g for 30 min at 4°C. The supernatant was collected and the protein concentration of the samples was determined in triplicates using Pierce BCA Protein Assay Kit (Thermo Scientific) and ELISA. Before separating proteins using SDS-PAGE, immunoprecipitation was done using 200 µg protein of each sample. Protein G beads (Invitrogen) were prewashed 3 times in PBS and 1 time in lysis buffer. Immune complexes were formed by adding 20 µg LINGO-1-ab (Novartis) to 100 µl beads followed by 2 h incubation at 4°C. The Protein G-ab immune complex was washed in lysis buffer, added to the cell lysates and incubated at 4°C over night. The protein G-ab-protein complexes were washed 2 times in lysis buffer and 1 time in PBS. The samples were boiled in 2×SDS Laemmli buffer (Sigma) at 95°C for 5 min before loaded on the SDS-PAGE gel (NuPAGE 4–12% Bis Tris Gel, Invitrogen). The gel was run at 200 V for 35 min in NuPAGE MES buffer (Invitrogen). The proteins were blotted using a PVDF filter (0.2 µm, Invitrogen) pretreated in methanol for 5 min and for 10 min in transfer buffer (Invitrogen). Transferring was done at 25 V, 120 mA for 100 min. The filter was blocked in 5% BSA in 0.2% Tween in PBS (PBS-T) for 60 min and then washed 2 times in 0.2% PBS-T. The filter was incubated with primary antibody (anti-LINGO-1-antibody, 0.4 mg/ml, Abcam) in 0.5% BSA and 0.2% PBS-T at 4°C over night. After washing, the filter was incubated with peroxidase-conjugated secondary antibody for 1 h at RT, washed in PBS-T and developed using enhanced chemiluminescence (ECL) system (GE Healthcare).

### Western blot analysis: Total lysate

Protein lysates were prepared by incubating the cells in lysis buffer (20 mM Tris pH 7.5, 0.5% Triton-X-100, 0.5% Deoxycholic acid, 150 mM NaCl, 10 m M EDTA, 30 mM NaPyroP) supplemented with 15% protease inhibitor cocktail (Roche) and sodium orthovanadate (Na_3_VO_4_) (0.1 M, Sigma) on ice for 30 min prior to 30 min centrifugation at 12000× g at 4°C. 30 µg protein was loaded on a SDS-PAGE gel (NuPAGE 4–12% Bis Tris Gel, Invitrogen) and the gel was run at 200 V for 90 min in NuPAGE MES buffer (Invitrogen). The proteins were blotted using a PVDF filter (0.2 µm, Invitrogen) pretreated in methanol for 5 min and for 10 min in transfer buffer (Invitrogen). Transferring was done at 25 V, 120 mA for 100 min. The filter was blocked in 5% BSA in 0.2% PBS-T for 60 min and then washed 2 times in PBS-T prior to incubation with primary antibody in 0.5% BSA and 0.2% PBS-T at 4°C over night. Primary antibodies were: rabbit-anti-PKB/c-Akt (Cell Signaling Technology), diluted 1∶1000 and rabbit-anti-phosphorylated PKB/c-Akt (Cell Signaling Technology), diluted 1∶1000. After washing, the filter was incubated with peroxidase-conjugated secondary antibody for 1 h at RT, washed in PBS-T and developed using enhanced chemiluminescence (ECL) system (GE Healthcare). Before rehybridization the filter was dehybridized in 0.4 M NaOH at RT for 10 min and washed 4×5 min in PBS-T.

### Statistics

The results are expressed as means ± standard error of the mean (sem). All data in the study were first tested for normality using Shapiro-Wilk test, showing non-normal distribution. The levels of significance were then determined by the non parametric Mann-Whitey tests (p<0.05 was designated with one asterisk, p<0.01 was designated with two asterisks and p<0.001 designated with three asterisks). All statistical analyses were performed using GraphPad Prism 5 (GraphPad Software, Inc., CA, USA).

## Supporting Information

Figure S1 **Two different LINGO-1 antibodies identify the same cells.** Double immunostainings show that the Novartis antibody and a LINGO-1 antibody purchased from Abcam identify the same LINGO-1 expressing cells. Differentiated NSPCs cultures were fixed at day 6 after mitogen withdrawal. Scale bars = 20 µm.
(TIF)

Figure S2 **Dose-Reponse effect of LINGO-1 neutralization during NSCP differentiation.** To investigate the effect of different concentrations of the LINGO-1 antibody on neuronal differentiation, NSPCs were cultured for 6 days in the presence of 1, 10, 100 or 1000 µg/ml LINGO-1 ab (Novartis) following mitogen withdrawal. Control cultures were left untreated or were treated with 100 µg/ml control, anti-lyszyme antibody. The cells were fixed and stained with specific antibodies against βIIItubulin. In untreated cultures and cultures treated with the control antibody, neurons were rather mature with multiple, long extending processes. Already in cultures treated with 1 or 10 µg/ml LINGO-1 antibodies the neurons had clearly a more immature phenotype. In cultures treated with 100 or 1000 µg/ml LINGO-1 antibody the difference to control cultures were more pronounced and the neurons in these cultures had very short processes. At the highest concentration the neurons were more often found in clusters. Scale bars = 20 µm.
(TIF)
