# Supplementary figures and images for: Neutralization of LINGO-1 during In Vitro Differentiation of Neural Stem Cells Results in Proliferation of Immature Neurons
Source: PLoS One. 2012 Jan 3;7(1):e29771. doi: 10.1371/journal.pone.0029771 (PMC3250485; doi:10.1371/journal.pone.0029771)

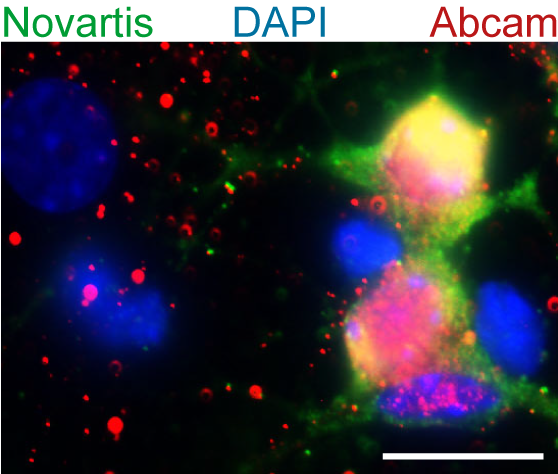

Supplement: Figure S1 — Two different LINGO-1 antibodies identify the same cells. Double immunostainings show that the Novartis antibody and a LINGO-1 antibody purchased from Abcam identify the same LINGO-1 expressing cells. Differentiated NSPCs cultures were fixed at day 6 after mitogen withdrawal. Scale bars = 20 µm. (TIF) [file pone.0029771.s001.tif]

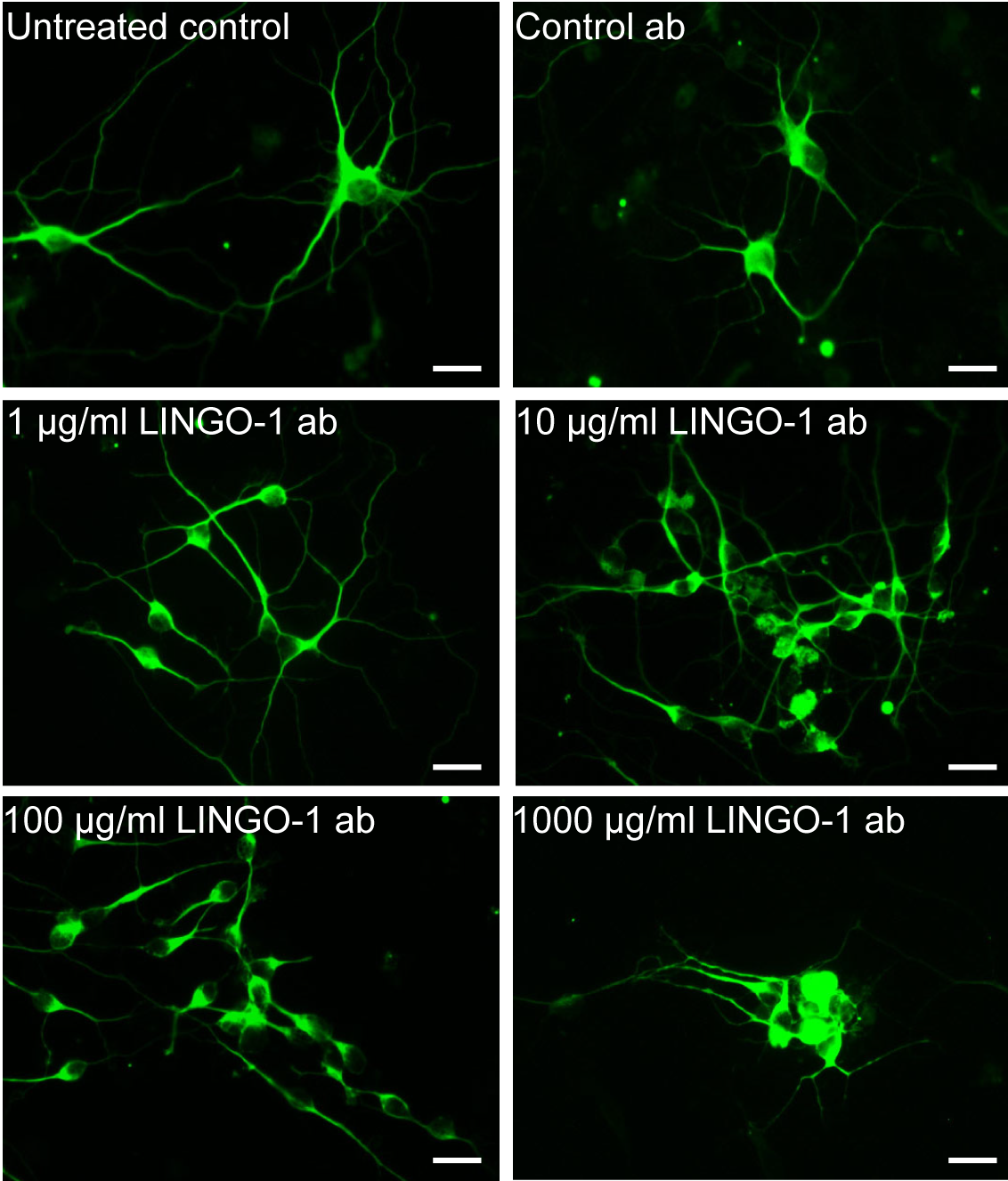

Supplement: Figure S2 — Dose-Reponse effect of LINGO-1 neutralization during NSCP differentiation. To investigate the effect of different concentrations of the LINGO-1 antibody on neuronal differentiation, NSPCs were cultured for 6 days in the presence of 1, 10, 100 or 1000 µg/ml LINGO-1 ab (Novartis) following mitogen withdrawal. Control cultures were left untreated or were treated with 100 µg/ml control, anti-lyszyme antibody. The cells were fixed and stained with specific antibodies against βIIItubulin. In untreated cultures and cultures treated with the control antibody, neurons were rather mature with multiple, long extending processes. Already in cultures treated with 1 or 10 µg/ml LINGO-1 antibodies the neurons had clearly a more immature phenotype. In cultures treated with 100 or 1000 µg/ml LINGO-1 antibody the difference to control cultures were more pronounced and the neurons in these cultures had very short processes. At the highest concentration the neurons were more often found in clusters. Scale bars = 20 µm. (TIF) [file pone.0029771.s002.tif]
